# Supplementary material for: URMAP, an ultra-fast read mapper
Source: PeerJ. 2020 Jun 24;8:e9338. doi: 10.7717/peerj.9338 (PMC7320720; doi:10.7717/peerj.9338)
Supplement: Table S1 — Speed is measured relative to BWA with file i/o overhead minimized. [file peerj-08-9338-s001.pdf]

Method	Speed
URMAPv	27.9
URMAP	10.3
Hisat2	9.5
SNAP	7.3
FSVA	5.4
Bowtie2	2.4
Minimap2	1.6
BWA	1.0
