# Supplementary material for: URMAP, an ultra-fast read mapper
Source: PeerJ. 2020 Jun 24;8:e9338. doi: 10.7717/peerj.9338 (PMC7320720; doi:10.7717/peerj.9338)
Supplement: Table S2 — Accuracy metrics are S (sensitivity) and E (error rate) with MAPQ ≥ 10, expressed as percentages. Superscript is var for the variant genome (NA12878) or ref for the reference (GRCh38), subscript is r for per-read or l for per-locus. [file peerj-08-9338-s002.pdf]

Method	$S^{var}{}_r$	$E^{var}{}_r$	$S^{var}{}_l$	$E^{var}{}_l$	$S^{ref}{}_r$	$E^{ref}{}_r$	$S^{ref}{}_l$	$E^{ref}{}_l$
BWA	96.3%	1.21%	97.4%	1.2%	96.7%	0.01%	97.3%	0%
Bowtie2	92.4%	0.09%	94.5%	0.059%	94.9%	0.02%	95.7%	0%
FSVA	88.9%	3.78%	94.0%	2.2%	93.8%	1.23%	96.1%	0.11%
Hisat2	89.9%	2.36%	93.6%	1.8%	95.5%	0.52%	97.0%	0.13%
Minimap2	91.0%	0.88%	93.8%	1.3%	93.7%	0.02%	95.5%	0%
SNAP	94.9%	1.25%	96.8%	1.1%	96.5%	0.12%	97.3%	0.026%
URMAP	95.2%	0.79%	96.8%	0.66%	96.1%	0.17%	96.8%	0%
URMAPv	90.0%	0.97%	92.7%	1.1%	93.7%	0.27%	95.2%	0.0055%
