# Supplementary material for: URMAP, an ultra-fast read mapper
Source: PeerJ. 2020 Jun 24;8:e9338. doi: 10.7717/peerj.9338 (PMC7320720; doi:10.7717/peerj.9338)
Supplement: Table S3 — Accuracy metrics are sensitivity and error rate with MAPQ ≥10, expressed as percentages. Species are Drosophila melanogaster (dm), Arabidopsis thaliana (at) and Homo sapiens (hs). [file peerj-08-9338-s003.pdf]

Length	Species	bowtie2	bwa	fsva	hisat2	minimap2	snap	urmap
150	at	93.8(0.33)	99.1(0.00)	92.3(0.22)	96.3(0.45)	99.0(0.00)	99.0(0.00)	98.4(0.00)
150	dm	91.2(0.47)	98.6(0.01)	88.9(0.30)	94.8(1.06)	97.8(0.01)	98.5(0.01)	97.7(0.03)
150	hs	90.4(0.28)	96.5(0.03)	88.2(0.61)	93.0(1.49)	95.5(0.02)	96.0(0.20)	95.0(0.80)
250	at	95.1(0.14)	99.4(0.00)	98.4(0.18)	90.0(0.51)	99.2(0.00)	98.9(0.00)	99.0(0.00)
250	dm	92.8(0.24)	99.6(0.00)	96.6(0.19)	89.8(1.14)	98.9(0.00)	99.0(0.00)	99.2(0.02)
250	hs	91.9(0.26)	97.1(0.01)	95.1(0.59)	88.0(1.80)	96.3(0.01)	96.4(0.11)	95.9(0.73)
300	at	95.4(0.11)	99.4(0.00)	98.8(0.17)	85.9(0.55)	99.0(0.00)	98.5(0.00)	99.0(0.00)
300	dm	93.2(0.21)	99.6(0.00)	97.7(0.17)	85.7(1.16)	98.7(0.02)	98.4(0.00)	99.3(0.02)
300	hs	92.5(0.24)	97.2(0.01)	95.9(0.61)	84.7(1.90)	96.1(0.03)	95.8(0.10)	96.1(0.72)
