# Supplementary material for: URMAP, an ultra-fast read mapper
Source: PeerJ. 2020 Jun 24;8:e9338. doi: 10.7717/peerj.9338 (PMC7320720; doi:10.7717/peerj.9338)
Supplement: Table S4 — For each method, the table shows the number of unmapped reads (i.e., reads with no reported alignment) and numbers of reads aligned with MAPQ=0, MAPQ=1, MAPQ ≥ 10 and total number of mapped reads (Total Mapped), i.e. aligned reads with MAPQ ≥ 0. [file peerj-08-9338-s004.pdf]

	Unmapped	MAPQ=0	MAPQ=1	MAPQ≥10	Total Mapped
Bowtie2	17,666,223	7,077,301	23,133,412	469,936,740	520,327,073
BWA	3,144,866	30,380,300	6,36,145	499,499,571	534,848,430
Hisat2	40,699,971	24,75,552	13,260,727	481,557,046	497,293,325
Minimap2	6,437,382	25,090,222	12,280,957	486,945,308	531,555,914
SNAP	21,840,285	12,214,496	6,402,678	487,784,019	516,153,011
URMAP	15,914,042	22,774,825	1,437,461	487,160,133	522,079,254
URMAPv	39,858,685	19,150,939	1,243,423	468,811,744	498,134,611
